# Perceptions of Breast Cancer Risks Among Women Receiving Mammograph Screening

**DOI:** 10.1001/jamanetworkopen.2022.52209

**Published:** 2023-01-23

**Authors:** Laura B. Beidler, Nancy R. Kressin, Jolie B. Wormwood, Tracy A. Battaglia, Priscilla J. Slanetz, Christine M. Gunn

**Affiliations:** 1 The Dartmouth Institute for Health Policy and Clinical Practice, Geisel School of Medicine, Dartmouth College, Hanover, New Hampshire; 2 Section of General Internal Medicine, Boston University Chobanian and Avedesian School of Medicine, Boston, Massachusetts; 3 Department of Psychology, University of New Hampshire, Durham; 4 Department of Radiology, Boston University Chobanian and Avedisian School of Medicine, Boston, Massachusetts; 5 Dartmouth Cancer Center, The Dartmouth Institute for Health Policy and Clinical Practice, Geisel School of Medicine, Dartmouth College, Hanover, New Hampshire

## Abstract

**Importance:**

Breast density is an independent risk factor for breast cancer. Despite the proliferation of mandated written notifications about breast density following mammography, there is little understanding of how women perceive the relative breast cancer risk associated with breast density.

**Objective:**

To assess women’s perception of breast density compared with other breast cancer risks and explore their understanding of risk reduction.

**Design, Setting, and Participants:**

This mixed-methods qualitative study used telephone surveys and semistructured interviews to investigate perceptions about breast cancer risk among a nationally representative, population-based sample of women. Eligible study participants were aged 40 to 76 years, reported having recently undergone mammography, had no history of prior breast cancer, and had heard of breast density. Survey participants who had been informed of their personal breast density were invited for a qualitative interview. Survey administration spanned July 1, 2019, to April 30, 2020, with 2306 women completing the survey. Qualitative interviews were conducted from February 1 to May 30, 2020.

**Main Outcomes and Measures:**

Respondents compared the breast cancer risk associated with breast density with 5 other risk factors. Participants qualitatively described what they thought contributed to breast cancer risk and ways to reduce risk.

**Results:**

Of the 2306 women who completed the survey, 1858 (166 [9%] Asian, 503 [27%] Black, 268 [14%] Hispanic, 792 [43%] White, and 128 [7%] other race or ethnicity; 358 [19%] aged 40-49 years, 906 [49%] aged 50-64 years, and 594 [32%] aged ≥65 years) completed the revised risk perception questions and were included in the analysis. Half of respondents thought breast density to be a greater risk than not having children (957 [52%]), having more than 1 alcoholic drink per day (975 [53%]), or having a prior breast biopsy (867 [48%]). Most respondents felt breast density was a lesser risk than having a first-degree relative with breast cancer (1706 [93%]) or being overweight or obese (1188 [65%]). Of the 61 women who were interviewed, 6 (10%) described breast density as contributing to breast cancer risk, and 43 (70%) emphasized family history as a breast cancer risk factor. Of the interviewed women, 17 (28%) stated they did not know whether it was possible to reduce their breast cancer risk.

**Conclusions and Relevance:**

In this qualitative study of women of breast cancer screening age, family history was perceived as the primary breast cancer risk factor. Most interviewees did not identify breast density as a risk factor and did not feel confident about actions to mitigate breast cancer risk. Comprehensive education about breast cancer risks and prevention strategies is needed.

## Key Points

**Question:**

How do women perceive the breast cancer risk associated with breast density, and how do they plan to mitigate their risk?

**Findings:**

In this qualitative study of women aged 40 to 76 years, family history was perceived as the greatest risk factor for breast cancer. In interviews, few women perceived breast density as a risk factor, and one-third thought that they could not take any actions to reduce their breast cancer risk.

**Meaning:**

Despite laws that require women to be notified about breast density, women did not describe a strong understanding of the risk associated with breast density relative to other breast cancer risk factors.

## Teaser

This qualitative study of women of breast cancer screening age examines how women perceived breast density as a cancer risk compared with other breast cancer risk factors.

## Introduction

Dense breasts, in which breasts are composed of more glandular tissue relative to fatty tissue, is an independent, nonmodifiable risk factor for breast cancer and can mask cancer on mammograms.^1^ Dense breast tissue is present in 40% to 50% of women undergoing screening mammography^2^ and is associated with a 1.2 to 4.0 times higher risk of breast cancer (depending on degree of density) compared with a 2.0 times higher risk associated with a first-degree family history of breast cancer.^3,4,5,6^ Other known risk factors include obesity, alcohol consumption, parity, and having a prior breast biopsy (eAppendix 1 in Supplement 1).^3,7,8^ Although how much each risk factor or combination of factors affects overall breast cancer risk has not been completely characterized,^7^ knowledge about personal risk is necessary to promote engagement in prevention, particularly for modifiable contributors, such as alcohol consumption and obesity.

Aiming to increase awareness and empower women to make informed choices about supplemental screening, laws enacted across 38 states mandate that women receive written notification about their personal breast density and its potential health implications.^9^ Although laws vary among states,^9^ they share an underlying goal of informing women about their personal breast cancer risk to promote informed decision-making about breast cancer screening and early detection.

Prior studies^10,11,12,13,14,15,16,17^ have evaluated the association of breast density notification laws with women’s awareness of their individual breast density, masking bias, and the risks associated with breast density. Qualitative studies have found that few women are aware of the legislation around breast density notification,^15^ that some women find breast density notifications to be confusing,^17^ and that, although most women understand that breast density could mask cancer on a mammogram, few know that breast density is an independent breast cancer risk.^13^ Cross-sectional surveys have found variation in women’s knowledge about breast density as a risk factor^10,11,12,14,16^; variation in knowledge across racial and ethnic groups, income, and educational levels^11,14^; that most women were aware of masking bias^11,14,16^; and that women in states that mandated breast density notification were more likely to report having dense breasts.^14^

Although the current literature explores women’s knowledge about breast density, a systematic review^18^ noted that little is known about whether women understand the risk associated with breast density compared with other risk factors or their approaches to mitigating risk. We used a national survey and qualitative interviews to examine how women perceive breast density’s cancer risk relative to other breast cancer risk factors and their understanding of actions they could take to reduce breast cancer risk.

## Methods

### Overview and Design

This mixed-methods qualitative study included survey data from a national, random-digit-dialing telephone survey coupled with semistructured interviews with a subset of survey respondents. Survey questions examined women’s perception of breast density in relation to other known breast cancer risks; interviews explored women’s understanding of breast cancer risk factors and actions to mitigate risk. This mixed-methods approach allowed us to examine the scope of awareness and understanding. On the basis of prior literature demonstrating differences in perceptions by sociodemographic characteristics,^11,14^ we examined whether risk perceptions varied by self-reported race and ethnicity and by literacy level (high literacy [HL] vs low literacy [LL]). This study was reviewed by the Boston University Medical Campus Institutional Review Board, which determined that the study met federal exemption criteria and provided a waiver of documentation of informed consent. Approval was for the qualitative interviews (survey work was conducted by an external survey firm) and at the time of transcription. All interview data was deidentified. The study followed the Standards for Reporting Qualitative Research (SRQR) for reporting qualitative data.^19^

### Setting and Sampling

The sampling frame consisted of 2306 participants who completed a national, random-digit-dialing survey of the effect of states’ breast density notification laws on knowledge about breast cancer risks associated with breast density. Eligible participants were aged 40 to 76 years, reported having undergone mammography in the prior 2 years, had no history of breast cancer, and had heard of breast density. Within the population-based sampling, efforts were made to ensure a sufficient sample of women from diverse racial and ethnic backgrounds, from states with and without breast density notification laws, and with lower literacy levels, as detailed in prior publications.^20,21^ Participants were asked in the survey to self-identify their race or ethnicity. We collected race and ethnicity data to allow for oversampling across some groups to ensure that we could conduct analyses that compared findings across groups.

After completing the survey, women who reported knowing their breast density were invited to participate in a qualitative interview. Those who responded affirmatively were called to schedule an interview. We purposively sampled equal numbers of women who identified as Black, Hispanic, White, or other race or ethnicity as well as those with HL vs LL. In the survey, participants were asked to self-identify their race from a list that included Asian, Black or African American, Native American, Pacific Islander, White, mixed race, or some other race. For these analyses, anyone who responded that they were Native American, Pacific Islander, mixed race, or some other race were classified as other race. For the qualitative interviews, we included respondents who were Asian in the other race category.

### Data Collection

Breast density awareness and breast cancer risk questions were adapted from measures used in prior surveys,^10,11,22^ with modified measures tested by patient advisory group members. Advisory group members also reviewed the interview guide. The survey firm, SSRS, conducted all surveys using a standardized interview approach (eAppendix 2 in Supplement 1). The cooperation rate for the overall survey was 85%.^20^ Survey administration spanned July 1, 2019, to April 30, 2020, and took approximately 10 minutes. Qualitative interviews were conducted from February 1 to May 30, 2020, and lasted 30 to 45 minutes. Qualitative interviews followed a flexible, semistructured interview guide (eAppendix 3 in Supplement 1) and were audiorecorded and transcribed. All data were collected via telephone by trained interviewers.

### Statistical Analysis

#### Survey

This mixed-methods qualitative study focused on women’s perceptions of breast cancer risks, examining how women rate certain risks relative to the risk of breast density. Women were asked to compare the risk of breast density with 5 other breast cancer risk factors (having a first-degree relative with breast cancer, being overweight or obese, having more than 1 alcoholic drink per day, never having children, or having a prior breast biopsy). A review of data from the first 448 survey participants revealed that wording of the risk perceptions questions was confusing. We revised the questions and excluded those participants from analyses due to identified measurement error and incompatibility of responses with subsequent risk questions. For each risk factor, participants were asked the question, “Which do you think puts someone at greater risk for developing breast cancer? Having dense breasts or…” Risk factors were elicited in a random order to minimize ordering bias.

We characterized the proportion of women who said having dense breasts puts someone at a greater risk for developing breast cancer vs the alternative risk factor or “don’t know”; participants with missing responses were excluded from analyses (<1%). Bivariate χ^2^ analyses assessed whether the proportion of women who said having dense breasts puts someone at greater risk for developing breast cancer was associated with participants’ race and ethnicity (coded as Asian, Black, Hispanic, White, and other category not listed) or literacy level (HL or LL). Low literacy was defined as either having less than a high school education or reporting sometimes, often, or always needing assistance to complete medical forms using the validated Single Item Literacy Screener.^23^ We used SPSS statistics software, version 26 (IBM Inc).^24^ Statistical significance was defined at α = .05. We followed the American Association for Public Opinion Research (AAPOR) reporting guidelines for survey data.^25^

#### Qualitative Interviews

Women were asked in an open-ended fashion what they thought contributed to breast cancer risk and how they could reduce their breast cancer risk. To organize and support analyses, we developed an analytic memo that described all observed themes.^26^ We used a matrix coding approach to guide development of themes and justify inclusion or exclusion of interviewees within themes.^27^ This approach includes arranging data within a table where individual participants represent rows and themes represent columns. We analyzed whether themes varied across literacy levels or across racial and ethnic groups. Qualitative analyses were overseen by a doctoral-level health services researcher (C.M.G.) with expertise in qualitative methods. Two masters-level trained research coordinators and 1 doctoral student participated in data collection and analysis, including co-coding and consensus determination meetings.

## Results

### Survey Data

Of the 2306 women who responded to the survey, 1858 (166 [9%] Asian, 503 [27%] Black, 268 [14%] Hispanic, 792 [43%] White, and 128 [7%] other race; 358 [19%] aged 40-49 years, 906 [49%] aged 50-64 years, and 594 [32%] aged ≥65 years) completed the revised risk perception questions and were included in the analysis (Table 1). In comparing risk factors with the risk associated with breast density, 1706 women (93%) viewed family history of breast cancer as the greater risk, and 1188 (65%) felt that being overweight or obese was a greater risk than breast density. Half of respondents thought that breast density was a greater risk than not having children (957 [52%]), having more than 1 alcoholic drink per day (975 [53%]), or having a prior breast biopsy (867 [48%]) (Figure). A higher proportion of women with LL compared with women with HL rated breast density as a higher risk than family history (13% vs 7%; χ^2^_1_ = 12.99, *P* < .001), alcohol consumption (60% vs 53%; χ^2^_1_* = *5.41, *P* = .02), and never having children (60% vs 51%; χ^2^_1_ = 7.39, *P* = .007). A higher proportion of Black women (290 [58%]) and Hispanic women (153 [58%]) rated dense breast as a higher risk than alcohol consumption compared with women of other races (χ^2^_4_ 13.63, *P* = .009). A total of 289 Black (58%) and 153 Hispanic (58%) women also rated dense breasts as a higher risk than nulliparity than women who identified as Asian (74 [45%]), White (377 [48%]), and other race (64 [52%]) (χ^2^_4_ = 17.48, *P* = .002).

**Table 1.** Survey and Interview Participant Characteristics

Characteristic	No. (%) of participants	
	Survey group (n = 1858)	Interview group (n = 61)
Race and ethnicity		
Asian	166 (9)^a^	NA^b^
Black	503 (27)^a^	14 (23)
Hispanic	268 (14)^a^	15 (25)
White	792 (43)^a^	17 (27)
Other^c^	128 (7)^a^	15 (25)^b^
Age, y		
40-49	358 (19)	8 (13)
50-64	906 (49)	24 (39)
≥65	594 (32)	18 (30)
Unknown	NR	11 (18)
Literacy^d^		
High	1560 (84)	33 (54)
Low	298 (16)	28 (46)
Residency in a state with an active breast density notification law	1403 (76)	50 (82)

Abbreviations: NA, not applicable; NR, none reported.

^a^ Data missing for 1 respondent.

^b^ Asian and other were collapsed into 1 category for qualitative interviews because of sample size constraints due to the goal of equal sampling across several groups.

^c^ Included women identifying as Native American, Pacific Islander, mixed race, or any other race not listed.

^d^ The low literacy group includes any participant who responded sometimes, often, or always on a single-item screener or who had less than a high school education.

**Figure.**
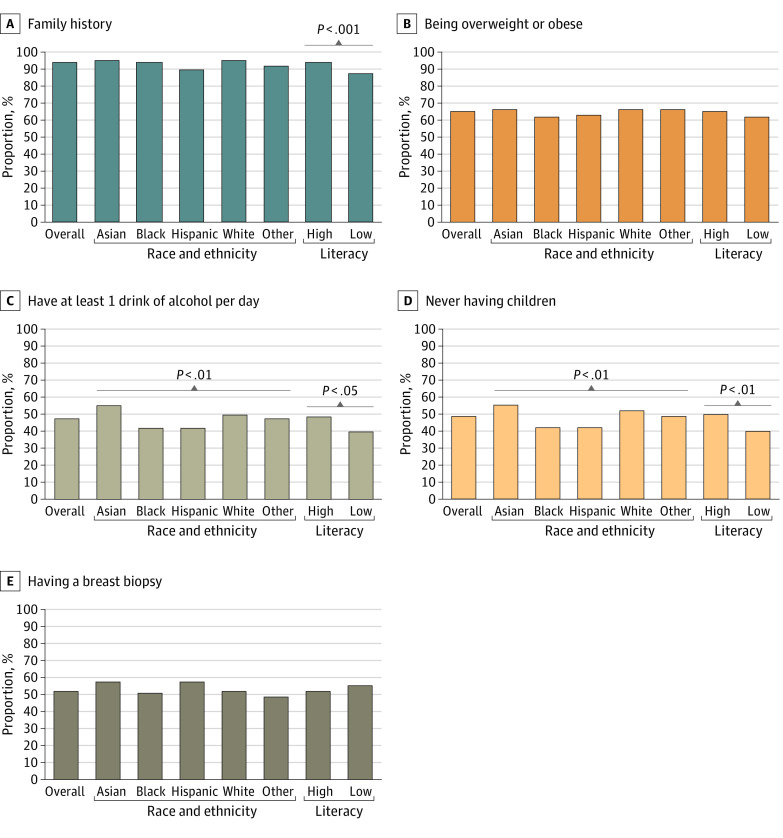
Women’s Perception of Comparative Risk of Breast Density With Other Known Breast Cancer Risk Factors The figure shows the percentage of women who answered that having the other risk factor put a woman at greater risk compared with dense breasts. Family history is defined as having a mother or sister who has or had breast cancer. Other race included women identifying as mixed race or another race or ethnicity. Data were missing for the following categories: being overweight or obese, 23; having 1 or more drinks of alcohol per day, 23; first-degree family history of breast cancer, 15; never having children, 27; having a breast biopsy, 32; and race and ethnicity, 1.

### Interview Data

#### Perceived Risk of Breast Cancer

Among 61 women interviewed, few women perceived breast density as contributing to their risk of developing breast cancer. Most women correctly noted that breast density could make mammograms harder to read: “It’s difficult to detect subsequent lumps or potential problem areas because of the dense breast tissue.” (Black woman, HL, respondent 7). When asked about their personal risk factors for breast cancer, few women noted that breast density could be a risk factor. One woman described her concern by saying, “Maybe 10% more worried than I was before because of the dense tissue issues. Just a slight uptick, but it’s not overwhelming” (Hispanic woman, HL, respondent 17).

Women most frequently and confidently emphasized family history of cancer or genetic factors as contributing to their own breast cancer risk (Table 2), and many viewed this as conferring very high levels of risk. One woman estimated her own risk as “probably 50/50 at this point since my mother had breast cancer” (Black woman, HL, respondent 5). Concurrently, women who had no known family history seemed to minimize the possibility of developing cancer: “I’m not worried about it because it does not run in my family. So I don’t have to worry about dodging that bullet” (Hispanic woman, LL, respondent 23).

**Table 2.** Perceived Breast Cancer Risk Factors, Ranked by Frequency of Mention by Interviewed Respondents

Perceived breast cancer risk factor	No. of respondents (n = 61)	Exemplar quotation (participant)
Family history and genetic factors	43	“Family, you know, people who’ve had it in your family. Like grandparents or as far back as, if every single generation of your family had it, you’re really at high risk. That is what, you know…I guess whoever had it before, you’re more, your chances are higher.” (Black woman, HL, respondent 3)
		“I’m not a carrier of the *BRCA* gene. I think that’s what [it’s] called.” (White woman, HL, respondent 46)
Diet	17	“And, not eating a healthy diet, not enough vegetables. Not enough fruits in a day, not enough fiber, proteins.” (woman of other race, LL, respondent 43)
		“And my diet wasn’t that great to be honest with you. I have a sweet tooth.” (White woman, LL, respondent 43)
Smoking	13	“I am concerned about, I should say that I am concerned about cancer because I smoke, and I know I need [to] stop smoking, and I have the [nicotine replacement] sitting right here on my table.” (Hispanic woman, LL, respondent 24)
Lifestyle (including stress, medication, and exercise)	13	“I’m very sedentary because of my job. I tend to be very much into, I stay long hours sitting, long hours and I know that it’s not good. And plus, I have to do a social job and I have sales, which is very stressful.” (Hispanic woman, LL, respondent 28)
Environmental factors	7	“But because of environmental aspects, I worked construction around a lot of different things.” (White woman, HL, respondent 45)
		“Maybe the air we breathe or pollution in the air and chemicals, ant spray, fly spray. Who needs it? I don’t know.” (White woman, LL, respondent 58)
Breast density	6	“My understanding is that breast tissue that is dense can be a higher indicator, or have a higher causative factor, toward breast cancer.” (Hispanic woman, HL, respondent 7)
Obesity	6	“I think obesity. I think your diet.” (Black woman, HL, respondent 1)
Alcohol	4	“Well, maybe the food that we eat, processed food, not exercising, not eating right. Those are the risks. Drinking, smoking, and just not taking care of herself.” (Black woman, LL, respondent 14)
		“Healthy weight, you know, drinking, like alcoholic beverages. I imagine that’s what it is. So drinking, exercise…eating habits, you understand?” (Hispanic woman, HL, respondent 15)
Reproductive history	4	“I have never been pregnant or given birth, so I know those are two things that weigh against me.” (White woman, HL, respondent 50)

Abbreviations: HL, high literacy; LL, low literacy.

Table 2 displays risk factors cited by women, ordered by the prevalence of the theme across participants. Reported risk factors included diet, lifestyle, smoking and environmental exposures, breast density, obesity, alcohol consumption, and reproductive history. Unlike family history, most women did not voice confidence in their understanding of other risk factors. Instead, they spoke about a series of behaviors and exposures that they perceived as related to their health overall: “We blame smoking for everything. So I’m sure smoking’s on there” (Black woman, HL, respondent 5). Few women stated that they had no knowledge of what breast cancer risk factors were: “I have no idea. All the stuff that’s been here on the news. This chemical, that chemical...” (Black woman, HL, respondent 8). We did not observe differences in understanding or perception of personal breast cancer risk by health literacy level or by racial or ethnic group.

#### Perceived Actions to Reduce Cancer Risk

When asked about actions that could reduce their breast cancer risk, many women described detection methods, such as breast self-examinations and mammograms, as prevention strategies. Among women who discussed mammograms or breast self-examinations, a small subset noted that screening methods would not prevent cancer but were useful for potentially detecting breast cancer earlier: “Well, if I go for my annual mammogram and do self-breast examination, I will catch whatever’s growing in my breast will be nipped in the bud...It will be taken care of before it gets out of control” (White woman, LL, respondent 54).

Women’s descriptions of risk mitigation focused on mammography, with descriptions conflating early detection and prevention. Other ideas for reducing personal breast cancer risk included improving diet, maintaining a healthy weight, quitting smoking, avoiding secondhand smoke, limiting alcohol, and exercising (Table 3). Many women suggested behaviors that they thought could improve their overall health but expressed less certainty about the direct effect on their breast cancer risk: “I try to eat a healthier lifestyle, more in the vegetable fields, less in any kind of…dairy or red meat portions. I do exercise more, but I did that for my general health, not for breast cancer” (Hispanic woman, HL, respondent 17).

**Table 3.** Perceived Actions to Reduced Breast Cancer Risk, Ranked by Frequency of Mention by Interviewed Respondents

Perceived action to reduce breast cancer risk	No. of respondents (n = 61)	Exemplar quotation (participant)
Timely mammograms	40	“But in addition to that, also doing the proper screenings every year, I think that that all plays an important part, you know, in keeping your risk of it down.” (Black woman, HL, respondent 1)
Improving or maintaining a healthy diet	32	“Well, I try to eat good nutrition food. I eat my vegetables and my fiber, and I try to stay away from sodas, and sugar, and high carb stuff, you know?” (Black woman, HL, respondent 2)
		“I have a 21-year-old son that keeps telling me that red meat and dairy products can trigger or can help to increase the possibilities of getting cancer. That’s why. That’s the only reason why I felt that eating milk and red meat, but I still eat cheese.” (Hispanic woman, LL, respondent 28)
Breast self-examinations	29	“I guess knowing how important the self-exams, self-breast exams you’re supposed to do every time you take a shower, you know, or whenever…I guess that’s real important to do. So, just to be real diligent and make sure you do that every month….So, just to see them and notice anything, you know, any difference or the way they feel or anything, inform somebody right away so they can look and find out what it is.” (Black woman, HL, respondent 3)
Exercise or active lifestyle (including maintaining a healthy weight)	20	“You just have to learn how to take care of yourself, and how to eat the right food, and how to exercise and keep yourself from getting overweight.” (woman of other race, LL, respondent 38)
		“And I think walking, just walking, it’s really good for the overall health. And so I try to walk as much as I can, meaning I walk on the treadmill, those types of things. So I guess just exercise, it’s helpful.” (woman of other race, LL, respondent 44)
Avoiding or quitting smoking	11	“Number one? I don’t smoke. I try to stay at my house. It’s a no smoking zone. I don’t even allow anyone to come in my driveway that smokes. Number two, I try to stay away from cigarette smoke.” (Black woman, LL, respondent 13)
Avoiding specific exposures (nuclear power plants, microwaves, chemicals, and bras with wires)	6	“I try to keep myself at a distance from microwave because every time I go close to microwave, I always think that I should stay away from it, but I haven’t stopped using it yet.” (woman of other race, HL, respondent 35)
		“I live my life the way I live it and I don’t think there is anything you can do other than stay away from nuclear plants or something like that. I don’t think there is anything you can do. It’s like the roll of the dice…you’re either lucky in life or you’re not lucky in life.” (Black woman, HL, respondent 6)
		“I wear a bra every day, so I don’t know if I didn’t wear a bra would that make it worse or...That’s what I’m saying. I don’t know.”(Hispanic woman, HL, respondent 7)
Avoiding or limiting alcohol	6	“Like I said, I feel if you’re going to get it, you’re going to get it. I think smoking and drinking and drugs, recreational drugs is something that might...It might add to it.” (Hispanic woman, HL, respondent 27)
Avoiding physical injury	2	“Avoiding injuries, unintentional injuries, realizing that it’s a very sensitive part of the body and being a little bit more careful.” (woman of other race, HL, respondent 34)

Abbreviations: HL, high literacy; LL, low literacy.

Many women (17 [28%]) stated that they were not sure if it was possible to reduce their breast cancer risk or that they did not know what actions they could take to reduce their risk: “Do people even know how to prevent breast cancer? I couldn’t even say” (woman of other race, HL, respondent 30). Neither health literacy level nor race or ethnicity appeared to differentiate how women perceived actions that they could take to reduce their breast cancer risk.

## Discussion

This mixed-methods qualitative study demonstrated that women perceived family history as the strongest risk factor for breast cancer, with mixed perceptions about other lifestyle or clinical risk factors in relation to breast cancer risk. Among interview respondents who knew their breast density, few women noted breast density as a breast cancer risk factor. Few women understood options for mitigating their personal breast cancer risk.

Despite breast density being associated with a 1.2 to 4 times higher risk of breast cancer,^1,5,6^ few women perceived breast density to be a strong personal risk factor. This finding is not surprising because prior studies^11,14^ have shown variable rates of women indicating that breast density contributed to breast cancer risk (23%-66%). Qualitative studies^13,17,28,29^ of women receiving breast density notifications found that women did not fully understand the clinical term *breast density*. It is possible that notification language stressing the normality of dense breast tissue in the population confers a sense of reassurance that may contribute to the downplaying of breast density as a risk factor.^13,29^

In both interviews and surveys, women perceived family history as highly deterministic of future breast cancer. Women without a family history believed they were safe or had limited risk based on this factor alone. Other studies^30,31^ have similarly found that women with family histories of breast cancer perceived their personal risk of cancer to be higher than the estimated risk associated with their family history. The emphasis on family history may be in part a result of clinical elicitation of family and genetic risk factors, including the increased emphasis on genetic testing for *BRCA1/2* genes, both clinically and in popular media.^32,33^ A 2021 systematic review^34^ found that in primary care, family history is often the only risk factor elicited to counsel patients on breast cancer risk. Thus, frequent health messaging around family history and breast cancer risk may play a role in how this sample of women perceived their own breast cancer risk. Interviewed women displayed little confidence in their ability to modify their cancer risk, suggesting a need for more comprehensive education about which risk factors are amenable to intervention.

Few women identified ways in which they could reduce their breast cancer risk. When mentioned, these actions included participating in regular screening, diet and exercise, and avoiding tobacco (Table 3). Many women suggested that breast self-examinations were important to maintaining their breast health, but these examinations are no longer recommended because of a lack of evidence of benefit.^35^^(p179)^^36^ Women also suggested actions that they thought were generally healthy lifestyle changes, but they were not confident these actions would alter their breast cancer risk. Women may benefit from general guidance and information about cancer prevention strategies, such as tools that can help patients understand overall cancer risk and prevention options.^37^ Clinical treatments, such as chemoprevention agents, are available to reduce breast cancer risk for women at elevated risk (>1.7% 5-year risk as determined by a validated risk model)^38,39^ but were not mentioned by any interviewees. This finding is not unexpected because chemoprevention is significantly underused by the eligible population,^40,41,42^ despite being recommended for women at elevated risk.^43^

### Limitations

This study has some limitations. Despite efforts to include a racially and ethnically diverse sample on the telephone survey panel, nonresponse bias could have influenced findings. The survey did not ask about women’s perception of the absolute risk associated with each risk factor, limiting our ability to draw conclusions about the accuracy of women’s risk perceptions. Interviewees reported being informed of their personal breast density, but we were unable to verify the nature or timing of this notification. We defined low literacy using a single-item literacy scale combined with educational level, which is an imprecise way to measure literacy, limiting our ability to draw conclusions about the direct effect of literacy on risk perception.

## Conclusions

Our study, coupled with prior research,^12,14,18,20^ suggests that understanding of breast density’s contribution to breast cancer risk remains underappreciated by many women. Most notifications encourage women to speak with their physicians, yet prior studies^15,44,45,46,47^ found that many clinicians do not feel comfortable counseling on the implications of breast density and cancer risk. Efforts to communicate breast density in part are intended to align with evidence suggesting that breast cancer screening services should be tailored to personal risk to maximize the benefits and avoid undue harms,^48,49,50^ rendering conversations about risk imperative. Women with dense breasts, and thus some elevated risk, are ideal candidates for risk assessment. Tools that incorporate breast density in risk measures, such as one from the Breast Cancer Surveillance Consortium,^51,52^ can inform future screening behaviors, including the opportunity for supplemental screening. Supplemental screening not only can lead to increased rates of cancer detection but also may result in more false-positive results and recall appointments.^53,54,55^ Because supplemental screening is not recommended for women at average risk,^55^ clinicians should use risk assessment to guide discussions with patients about tradeoffs associated with supplemental screening.

Despite available guidance on breast cancer risk assessment to inform screening decisions,^56,57^ such assessments are underused in primary care.^58,59,60^ Reported barriers include inadequate time, lack of integrated tools, and uncertainty in interpreting results for decision-making.^58^ A review^61^ of interventions involving the use of risk assessment tools in primary care concluded that more comprehensive interventions that combined risk assessment with decision support were more likely to have an effect on behavior. In some cases, it may be beneficial to develop partnerships between primary care and radiology to help counsel women on appropriate supplemental screening and/or preventive measures.^62^ In summary, future laws or regulations involving breast density notifications should ensure that communications promote a more comprehensive understanding of breast cancer risk to inform choices about screening and prevention.

## Notes

Supplement 1. **eAppendix 1.** Breast Cancer Risk Factors **eAppendix 2.** Survey Instrument Content **eAppendix 3.** Interview Guide

Supplement 2.
Data Sharing Statement
