# Supplementary material for: Perceptions of Breast Cancer Risks Among Women Receiving Mammograph Screening
Source: JAMA Netw Open. 2023 Jan 23;6(1):e2252209. doi: 10.1001/jamanetworkopen.2022.52209 (PMC9871800; doi:10.1001/jamanetworkopen.2022.52209)
Supplement: Supplement 1. — eAppendix 1. Breast Cancer Risk Factors eAppendix 2. Survey Instrument Content eAppendix 3. Interview Guide [file jamanetwopen-e2252209-s001.pdf]

Supplementary Online Content

Beidler LB, Kressin NR, Wormwood JB, Battaglia TA, Slanetz PJ, Gunn CM. Perceptions of breast cancer risks among women receiving mammograph screening. *JAMA Netw Open*. 2023;6(1):e2252209. doi:10.1001/jamanetworkopen.2022.52209

eAppendix 1. Breast Cancer Risk Factors
eAppendix 2. Survey Instrument Content
eAppendix 3. Interview Guide

This supplementary material has been provided by the authors to give readers additional information about their work.

© 2023 Beidler LB et al. *JAMA Network Open.*

**eAppendix 1.** Breast Cancer Risk Factors		
**Risk Factor**	**Comparison Group**	**Associated Risk of Invasive Breast Cancer**
1st degree relative with breast cancer	No 1st degree relative with breast cancer	RR 1.5 (95% CI 1.42,1.51)[1]<br>*Pre-menopausal:*<br>OR 1.71 (95% CI 1.59-1.84)[2]<br>*Post-menopausal*:<br>OR 1.53 (95% CI 1.46-1.60)[2]
Breast density	Scattered fibroglandular densities	*Pre-menopausal:*<br>almost entirely fat OR 0.47 (95% CI 0.38-0.58)<br>heterogeneously dense OR 1.57 (95% CI 1.46-1.69)<br>extremely dense OR 1.81 (95% CI 1.65-1.99)[2]<br>*Post-menopausal:*<br>almost entirely fat OR 0.62 (95% CI 0.58-0.67)<br>heterogeneously dense OR 1.40 (95% CI 1.34-1.45)<br>extremely dense OR 1.58 (95% CI 1.46-1.71)[2]
Overweight/obese	Normal weight	*Pre-menopausal:*<br>underweight OR 0.93 (0.76-1.15)<br>overweight OR 0.99 (0.93-1.07)<br>obesity class I OR 1.00 (0.91-1.10)<br>obesity class II OR 1.05 (0.94-1.18)[2]<br>*Post-menopausal:*<br>underweight OR 0.79 (95% CI 0.67-0.93)<br>overweight OR 1.23 (95% CI 1.17-1.28)<br>obesity class I OR 1.39 (95% CI 1.31-1.47)<br>obesity class II OR 1.54 (95% CI 1.45-1.64)[2]
Never having children	Having children	RR 1.23 (95% CI 1.12, 1.35)[1]
	Having first child at age 30 or younger	*Pre-menopausal:*<br>OR 1.14 (95% CI 1.05-1.22)[2]<br>*Post-menopausal:*<br>OR 1.20 (95% CI 1.14-1.26)[2]
Having one or more alcoholic drinks per day	0 g alcohol/day	*15+ g alcohol/day:*<br>RR 1.32 (95% CI 1.22, 1.42)[1]
Having a prior breast biopsy	No history of benign breast disease	*Benign breast disease*<br>RR 1.45 (95% CI 1.39, 1.51)[1]
	No history of benign breast biopsy	*Pre-menopausal benign breast biopsy:*<br>OR 1.5 (95% CI 1.40-1.62)[2]<br>*Post-menopausal benign breast biopsy:*<br>OR 1.41 (95% CI 1.35-1.47)[2]

Notes: RR = relative risk, OR = odds ratio, CI = confidence interval

© 2023 Beidler LB et al. *JAMA Network Open.*

eReferences

1. Tamimi RM, Spiegelman D, Smith-Warner SA, et al. Population Attributable Risk of Modifiable and Nonmodifiable Breast Cancer Risk Factors in Postmenopausal Breast Cancer. *American Journal of Epidemiology*. 2016;184(12):884-893. doi:10.1093/aje/kww145

2. Engmann NJ, Golmakani MK, Miglioretti DL, Sprague BL, Kerlikowske K, for the Breast Cancer Surveillance Consortium. Population-Attributable Risk Proportion of Clinical Risk Factors for Breast Cancer. *JAMA Oncology*. 2017;3(9):1228-1236. doi:10.1001/jamaoncol.2016.6326

© 2023 Beidler LB et al. *JAMA Network Open.*

eAppendix 2. Survey Instrument Content

[NOTE: *These questions are a subset of a larger survey. Only the survey items that pertain to the present report are included below. Thus, some of the skip patterns refer to items not included below, and the item numbers are not all sequential. The text below also includes instructions to the telephone interviewers who administered the survey (IN PARENTHESES AND CAPS).]*

BREAa1. Have you had a mammogram in the past 2 years?

1 Yes (CONTINUE)
2 No (STOP)
9 (DO NOT READ) Refused

BREAa2. Have you ever been told by a doctor or other healthcare provider that you have breast cancer?

(INTERVIEWER: IF RESPONDENT SAYS THEY WERE DIAGNOSED WITH DCIS (Ductal Carcinoma in Situ) OR LCIS (Lobular Carcinoma in Situ), CODE AS ‘YES’)

1 Yes (STOP)
2 No (CONTINUE)
9 (DO NOT READ) Refused (STOP)

BREAa3. Before today, had you ever heard of the terms ‘breast density’ or ‘dense breasts’?

1 Yes (CONTINUE)
2 No (STOP)
8 (DO NOT READ) Don’t know (STOP)
9 (DO NOT READ) Refused (STOP)

S0. And to ask you the right questions, can you tell me what state do you live in?______

© 2023 Beidler LB et al. *JAMA Network Open.*

BREA4. Have you ever received information about your **personal** breast density?

1 Yes
2 No (GO TO BREA6a)
8 (DO NOT READ) Not sure
9 (DO NOT READ) Declined to answer

BREA14. Several factors increase the risk of breast cancer. Which do you think puts someone at a greater risk for developing breast cancer? Having dense breasts or (INSERT ITEM)?

1 Having dense breasts
2 INSERT ITEM
8 (DO NOT READ) Not sure
9 (DO NOT READ) Declined to answer

(SCRAMBLE ROTATE)
a. being overweight or obese
b. having more than one drink of alcohol per day
c. having a mother or sister who has or had breast cancer
d. never having children
e. having had a breast biopsy

PERSONAL HEALTH LITERACY LEVEL

BREA19. How often do you need to have someone help you when you read instructions, pamphlets, or other written materials from your doctor or pharmacy? (READ LIST)

(ROTATE 1-5/5-1)

© 2023 Beidler LB et al. *JAMA Network Open.*

1 Never
2 Rarely
3 Sometimes
4 Often
5 Always
8 (DO NOT READ) Not sure
9 (DO NOT READ) Declined to answer

Z-7 What is your age?
(RECORD 2-DIGIT NUMBER)

____
99 Refused

(ASK Z-7a IF Z-7=REFUSED)
Z-7a Could you please tell me if you are ...?
(PHONE ONLY)(READ LIST)

0 Under 18 (TERMINATE IF CELL)
1 18-29
2 30-39
3 40-49
4 50-64
5 65-76
6 77+
9 (DO NOT READ) Refused

Z-10. Are you of Hispanic or Latino origin or descent?

1 Yes
2 No
8 Don't Know
9 Refused

© 2023 Beidler LB et al. *JAMA Network Open.*

RT-01. Do you consider yourself white, black or African American, Asian, Native American, Pacific Islander, mixed race or some other race? (ENTER ONE ONLY)

(IF RESPONDENT SAYS HISPANIC ASK: Do you consider yourself a white Hispanic or a black Hispanic?)

(INTERVIEWER NOTE: CODE AS WHITE (1) OR BLACK (2). IF RESPONDENTS REFUSED TO PICK WHITE OR BLACK HISPANIC, RECORD HISPANIC AS "OTHER,"

If "other" say: "I'm not referring to your nationality. I just want to know if you consider yourself white or black."

If respondent won't pick one, then enter code for "OTHER"

1 White
2 Black or African American
3 Asian/Chinese/Japanese
4 Native American/American Indian/Alaska Native
5 Native Hawaiian and Other Pacific Islander
6 Mixed
0 Other (SPECIFY)__________
9 Refused

Z-8. What is the highest level of school you have completed or the highest degree you have received? (DO NOT READ LIST)

(INTERVIEWER NOTE: Enter code 3-HS grad if Respondent completed training that did NOT count toward a degree)
(INTERVIEWER NOTE: Enter code 3-HS graduate if Respondent completed vocational, business, technical, or training courses after high school that did NOT count toward an associate degree from a college, community college or university (e.g., training for a certificate or an apprenticeship))

01 Less than high school (Grades 1-8 or no formal schooling) (OLD CODE 1)

© 2023 Beidler LB et al. *JAMA Network Open.*

02 High school incomplete (Grades 9-11 or Grade 12 with NO diploma) (OLD CODE 1)
03 High school graduate (Grade 12 with diploma or GED certificate) (OLD CODE 2)
04 Some college, no degree (includes community college) (OLD CODE 3)
05 Two-year associate degree from a college or university (OLD CODE 3)
06 Four-year college or university degree/Bachelor’s degree (e.g., BS, BA, AB) (OLD CODE 4)
07 Some postgraduate or professional schooling, no postgraduate degree (OLD CODE 5)
08 Postgraduate or professional degree, including master’s, doctorate, medical or law degree (e.g., MA, MS, PhD, MD, JD) (OLD CODE 5)
98 (DO NOT READ) Don’t Know
99 (DO NOT READ) Refused

© 2023 Beidler LB et al. *JAMA Network Open.*

eAppendix 3. Interview Guide

Qualitative Interview Guide (***NOTE: NOT ALL ITEMS/CONCEPTS ARE INCLUDED IN THE PRESENT PAPER)***

Please note: This is an interview guide, intended to be used flexibly, to allow for a conversational flow to the interview while covering the topics below. Prompts are included here as possible suggestions for elaboration if responses are short.

Thank you for talking to me today.

Today we will talk about two main topics: 1) Your experience learning about dense breasts; and 2) How breast density notifications could be improved to help women.

There are no right or wrong answers to the questions I'm going to ask today, I really just want to hear your opinion and thoughts. I understand that this that subject might be delicate and that it is personal. So, if you feel uncomfortable with the questions I ask, please say so and we can move on to another question or stop the interview entirely.

Do you have any questions before we get started?

TOPIC 1: LEARNING ABOUT DENSE BREASTS

Concept	Questions	Prompts
**Learning about Dense Breasts**	Tell me how you first learned that you had dense breasts.	Probe for:<br>Who gave you that message?<br>What did the letter/they say?<br><br>When in the mammogram process did you get this information?
	Thinking back, what was the main message that you took away from learning you had dense breasts?	
**Reaction/ Feelings**	What was your first reaction when you first learned that you had dense breasts?	• What was it like for you?<br>• How did it feel to learn that information?
	When you [had that reaction], what did you do?	• Who did you share the results with? What made you turn to them?<br>• How did sharing results go?
**Personal Meaning**	What does it mean to you that you have dense breasts?	• What is the purpose of telling women they have dense breasts?<br>• What makes your breasts different than other women's breasts?<br>• What do you think you are expected to do about that?<br>• Where you informed of next steps?
**Information Seeking**	Where did you go to find more information about breast density, if you did at all?	What information or sources did you find most useful?<br><br>What was made clear from these other sources?

		What information didn't get answered by these materials?
	Who have you talked to about your breast density?	• When did these conversations happen?<br>• What did you discuss?<br>• What changed for you as a result of those conversations?<br>• What did you learn from these conversations?<br><br>• *If no one*: Who would you have liked to talk to?<br>• Why haven't you talked to anyone?
**Action and Importance of Breast Health**	Now that you know your breast density, what do you feel is important for you to do to maintain your breast health?	• How did you decide [to do this]?<br>• Who did you consult in forming this plan?<br>• What information was most important to you in deciding on [action]?

TOPIC 2: BARRIERS AND FACILITATORS TO INFORMATION ABOUT BREAST DENSITY

Concept	Questions	Prompts
**Personal experience**	What do you think about the way you learned about your breast density?<br><br>What was the most difficult part about receiving your breast density results?	How informative was it?<br>How helpful was it?<br>What would you change about it?
**Improvements to the process**	How would you have liked to have received your breast density results, if any other way?<br><br>What information do you think patients need when they first learn about their own breast density?<br>• What else should women know that they aren't currently being told?<br><br>What could help women better understand their breast density results?	• Why?<br><br>• When should they learn about breast density?<br>• How?<br>• Who should deliver that message?<br><br>What has helped you better understand your breast density results, if anything?<br>• How has this helped you?

© 2023 Beidler LB et al. *JAMA Network Open.*

TOPIC 3: GENERAL BREAST HEALTH QUESTIONS

Concept	Questions	Prompts
**Priority**	How worried are you about getting breast cancer in the future?	What things do you think make you at higher/lower risk for breast cancer?<br><br>What actions do you take to reduce the chances you'll get breast cancer, if anything?
	In general, what health issues are of most importance to you?	How do mammograms and breast cancer rank in relation to these other concerns?

Conclusion

Those are all the questions I have for you today. Before we end, is there anything I have missed that you would like to share about the topics we have talked about today?

© 2023 Beidler LB et al. *JAMA Network Open.*
