# Supplementary material for: Perceptions of Breast Cancer Risks Among Women Receiving Mammograph Screening
Source: JAMA Netw Open. 2023 Jan 23;6(1):e2252209. doi: 10.1001/jamanetworkopen.2022.52209 (PMC9871800; doi:10.1001/jamanetworkopen.2022.52209)
Supplement: Supplement 2. — Data Sharing Statement [file jamanetwopen-e2252209-s002.pdf]

Data Sharing Statement

Beidler. Perceptions of Breast Cancer Risks Among Women Receiving Mammograph Screening. *JAMA Netw Open*. Published January 23, 2023.
doi:10.1001/jamanetworkopen.2022.52209

Data
Data available: No
